# Alpha-pinene moderates memory impairment induced by kainic acid via improving the BDNF/TrkB/CREB signaling pathway in rat hippocampus

**DOI:** 10.3389/fnmol.2023.1202232

**Published:** 2023-06-30

**Authors:** Paria Hashemi, Shamseddin Ahmadi

**Affiliations:** Department of Biological Science, Faculty of Science, University of Kurdistan, Sanandaj, Iran

**Keywords:** neuroprotective agent, CA1 region, working memory, avoidance learning, neurotrophin, CREB

## Abstract

**Introduction:**

The potential benefits of natural ingredients in the alleviation of neurodegenerative disorders are of great interest. Alpha-pinene (APN) is an essential oil belonging to monoterpenes with multiple beneficial effects. In this study, the possible improving effects of alpha-pinene on memory impairment induced by kainic acid and the underlying molecular mechanisms were examined.

**Methods:**

Memory impairment was induced by i.c.v. injection of kainic acid (KA) in male Wistar rats. Alpha-pinene (50 mg/kg/day, i.p.) was injected for 21 days, including 14 days before the KA injection and seven days afterward. Spatial working memory and inhibitory avoidance (IA) memory performance were assessed five and even days following KA injection, respectively. The hippocampal protein levels of brain-derived neurotrophic factor (BDNF), tropomyosin-like receptor kinase B (TrkB), cAMP response element binding protein (CREB), and neuronal loss in the CA1 region were also examined.

**Results:**

Results revealed that the i.c.v. injection of KA triggered memory impairment, which was notably diminished by alpha-pinene pre-and post-treatment. Histopathological evaluation revealed that alpha-pinene significantly moderated the attenuation in CA1 alive neurons induced by KA injection. Western blotting analysis confirmed that alpha-pinene pre-and post-treatment significantly reversed the KA-induced decreases in the hippocampal levels of BDNF, TrkB, phosphorylated TrkB, CREB, and phosphorylated CREB.

**Discussion:**

These findings suggest that alpha-pinene pre-and post-treatment moderate memory impairment induced by KA by restoring the BDNF/TrkB/CREB signaling pathway in the rat hippocampus.

## 1. Introduction

The ability of humans to perform daily life activities and proper functions in society is centrally dependent on memory (Khan et al., 2014). Memory impairment can result from brain damage due to trauma, stress, stroke, or epilepsy (Samuelson, 2011; Al-Qazzaz et al., 2014; Rayner et al., 2016). An epileptic seizure can potentially affect memory, either during or after the seizure (Tramoni-Negre et al., 2017). Based on accumulating clinical studies, learning, and memory deficits are among the most frequent cognitive declines in patients with temporal lobe epilepsy (TLE; Butler and Zeman, 2008; Celiker Uslu et al., 2019). The hippocampus, the main structure of the medial temporal lobe, is predominantly involved in recurrent spontaneous seizures originating from the temporal lobe (Butler and Zeman, 2008). Besides, it is well-documented that the hippocampus plays a critical role in learning and memory processes (Bartsch and Arzy, 2014). Increasing data also indicate that progressive neurodegeneration in the hippocampus following prolonged seizure activity correlates with impairments in learning and memory performance (Hashemi et al., 2019). Therefore, it is logical to hypothesize that moderating neurodegeneration in the hippocampus has a preventive effect on learning and memory impairments induced by seizures.

Neurotrophic factors include several families of growth factors with prominent functions in brain health and disease (Chao et al., 2006). Brain-derived neurotrophic factor (BDNF) is a neurotrophin involved in a variety of processes in developing and adult mammalian brains, including neuronal differentiation, axonal outgrowth, synaptic transmission, neuroprotection, modulation of synaptic plasticity, and higher cognitive functions [for review see (Park and Poo, 2013; Kowiański et al., 2018)]. The effects of BDNF on target cells are mediated by its strong affinity for tropomyosin-like receptor kinase B (TrkB) (Colucci-D’Amato et al., 2020). BDNF also plays a pivotal role in modulating memory formation (Bekinschtein et al., 2014). The prominent role of BDNF in memory formation and other cognitive functions is corroborated by the high expression of TrkB receptors in the hippocampus (Muragaki et al., 1995). Cyclic AMP (cAMP) response element-binding protein (CREB) is a nuclear transcription factor involved in various physiological processes, including synaptic plasticity, learning, and memory (Nair and Vaidya, 2006). CREB is a neuronal activity-dependent protein that plays a key role in hippocampal-dependent memory formation (Sen, 2019). Studies have shown that different agents that increase CREB activity can improve learning and memory function (Tully et al., 2003; Zarneshan et al., 2022). There are several reports on the interconnected functions of BDNF and CREB. For example, it has been shown that CREB mediates the effects of BDNF on dendritic growth (Finsterwald et al., 2010). Animal studies have also emphasized that the upregulation of BDNF and CREB proteins in the hippocampus improves memory impairment in experimental epilepsy models (Sharma et al., 2020).

The potential benefits of natural ingredients in the alleviation of neurodegenerative disorders are of great interest. Alpha-pinene (APN) is an essential oil belonging to monoterpenes with multiple beneficial effects, including antioxidative (Khan-Mohammadi-Khorrami et al., 2022), anti-inflammatory and antiapoptotic (Khoshnazar et al., 2020), antiseizure (Hashemi and Ahmadi, 2023), sedative, and anxiolytic properties (Khan-Mohammadi-Khorrami et al., 2022). Studies have shown that APN improves avoidance memory and motor activity in a rat model of Parkinson’s disease via neuroprotective effects against 6-hydroxy dopamine toxicity and by reducing oxidative damage (Goudarzi and Rafieirad, 2017). It has also been reported that APN inhalation enhances BDNF gene expression in the olfactory bulb and hippocampus in mice (Kasuya et al., 2015). APN also improved learning and memory performance in scopolamine-induced memory impairment in C57BL/6 mice (Lee et al., 2017). We recently reported that APN pretreatment for 2 weeks has an anti-seizure effect against KA-induced TLE. However, the possible beneficial effects of APN on KA-induced learning and memory decline and the underlying molecular mechanisms have not been investigated. Therefore, it would be logical to investigate the effects of APN on memory performance in a rat model of kainite-induced epilepsy.

Investigations of animal models for memory impairment have disclosed not only valuable information about the organization of memory in the brain but also provided solutions for more efficient control of the progression of memory loss. Kainic acid (KA), via binding to the kainate subtype of glutamate receptors, induces histopathological and behavioral alterations as well as learning and memory impairment in rodents, which is very similar to that seen in patients with TLE (Lévesque and Avoli, 2013; Jefferys et al., 2016). A common comorbidity of epilepsy is learning and memory impairments in patients with TLE (Xing et al., 2019). Therefore, the current study was designed to evaluate the possible positive effects of the APN pre-and post-treatment on KA-induced impairment of learning and memory performance in rats. The possible involvement of the BDNF/TrkB/CREB signaling pathway in the hippocampus was also examined.

## 2. Materials and methods

### 2.1. Animals

Forty male Wistar rats (200–250 g) were obtained from an animal laboratory colony at the University of Kurdistan. The rats were housed in four per cage under standard conditions, including a 12-h light/dark cycle (lights on at 7:00 AM), 22 ± 2°C, and 40–50% humidity. The animals had free access to food and water, except during the experiments. All procedures in this study followed the Guidelines for the Care and Use of Laboratory Animals (2011), defined by the National Academy of Sciences Institute for Laboratory Animal Research. The study protocol was approved by the Ethics and Research Committee of the University of Kurdistan (IR.UOK.REC.1400.024).

### 2.2. Treatments and experimental groups

Kainic acid was purchased from Sigma (Sigma-Aldrich Co., United States), and dissolved in ice-cold normal saline immediately before use. Alpha-pinene (APN) is a chemical constituent of the essential oils extracted from various plants, including conifers, wild pistachio, rosemary, and sage (Salehi et al., 2019). It is a colorless, water-insoluble, oil- and ethanol-soluble organic liquid. The APN oil used in this study was a gift from Van Company (Sanandaj, Iran), and was extracted from *Pistacia atlantica* subsp. kurdica (wild pistachio tree) with 97% purity. Therefore, the main component of the oil responsible for its effects is APN, which has the chemical formula C10H16. Apart from APN, other constituents such as beta-pinene and limonene may also be found in APN oil, but the quantities of these components are very low and often work synergistically, exerting combined effects that contribute to the overall neuroprotective potential of APN oil (Mahjoub et al., 2018). APN was diluted in 5% Dimethyl sulfoxide (DMSO; Merck Co., Germany) before use. A single dose of KA was intracerebroventricularly (i.c.v.) administered to induce memory impairment due to damage to the medial temporal lobe structures, mainly the hippocampus. The sham group received an i.c.v. injection of saline, instead of KA. APN was injected intraperitoneally (i.p.) once a day for 21 days at a dose of 50 mg/kg from 14 days before until 7 days following the i.c.v. injection of KA. The dose of APN was based on a recent report by other investigators and a recent report from our laboratory (Khan-Mohammadi-Khorrami et al., 2022; Hashemi and Ahmadi, 2023). DMSO (5%) was used as the vehicle for APN. Forty rats were randomly distributed into five experimental groups (*n* = 8 per group) as follows: (1) the control group with neither pre-and post-treatment of APN nor stereotaxic surgery; (2) DMSO + sham group, which received i.p. injections of DMSO and an i.c.v. injection of saline; (3) APN + sham group, which received i.p. injections of APN and an i.c.v. injection of saline; (4) DMSO + KA group, which received i.p. injections of DMSO and an i.c.v. injection of KA; and (5) APN + KA group, which received i.p. injections of APN and an i.c.v. injection of KA. Random distribution was performed using Random Allocation Software V1.0 (Saghaei, 2004).

### 2.3. Stereotaxic surgery

On day 14 of DMSO or APN pre-treatment, rats (except for the control group) were anesthetized with an i.p. injection of a mixture of ketamine (100 mg/Kg, i.p.) plus xylazine (10 mg/Kg, i.p.) and positioned in a stereotaxic frame (Stoelting Co., United States). The coordinates for the left lateral ventricle were as follows: AP, −1 mm relative to the bregma; 1.5 mm from the midline, and −3.5 mm beneath the dura (Paxinos and Watson, 2007). During the stereotaxic surgery, rats in DMSO + KA and APN + KA groups received an i.c.v. injection of 0.5 μg KA dissolved in 1.2 μl of normal saline by using a Hamilton syringe. Rats in the DMSO + Sham and APN + sham groups received i.c.v. injections of saline without KA (Hashemi and Ahmadi, 2023).

### 2.4. Y Maze task

Five days after the i.c.v. injection of saline or KA, spatial working memory was evaluated in a single-session Y-maze. Spontaneous alternations were assessed in a Y-shaped apparatus composed of a gray-color Plexiglas with three equal-sized arms named A, B, and C (50 × 10 × 40 cm height) interconnected by a triangular central arena. Each rat was placed at the end of the A-arm and allowed to explore the maze arms freely for 10 min. The animals’ behavior was videotaped and evaluated later by an experimenter who was blinded to the treatments. Spontaneous alteration behavior was defined as sequential entry into all three arms in overlapping triplet sets. The arm entries were counted when the animal entered the arm with all four paws. The maze arms were cleaned between trials with ethanol 10% to eliminate residual odor signs. The percentage (%) of spontaneous alternation behavior was calculated using the following formula:


$$\begin{array}{l} \text{Alternation}\\ \text{percentage} \end{array}=\left[\left(\begin{array}{l} \text{total number}\\ \text{of alternations} \end{array}\right)/\left(\begin{array}{l} \text{total number}\\ \text{of}\;\text{arm}\;\text{entries}-2 \end{array}\right)\right]\times 100.$$

### 2.5. Inhibitory avoidance task

A step-through inhibitory avoidance (IA) test was also carried out during 2 days after the Y Maze test. The IA apparatus was composed of two equal-sized white and black chambers (20 × 20 × 30 cm height) interconnected by a middle door (7 × 9 cm), which could be lifted manually. The walls and floor of the white chamber were made of white Plexiglass, but the walls of the black chamber consisted of black Plexiglass and its floor was made of stainless-steel bars 3 mm in diameter and located 1 cm apart from each other. In the acquisition phase, each rat was first placed in the white compartment, and the door between the two compartments was opened 5 s later. When the animal entered the black chamber, the middle door was closed and an electrical foot shock (1 mA, 3 s) was delivered to the stainless-steel rods by using an isolated stimulator (Borj Sanat Azma, Tehran, Iran). In the acquisition phase, the initial latency to enter the black compartment was recorded. Twenty-four hours following the acquisition phase, each rat was transferred to the white compartment for the memory recall test. After opening the middle door during the recall phase, the time spent in the white compartment before entry into the black compartment was recorded as step-through latency. A higher step-through latency indicates more memory of the shock delivered to the animal during the acquisition trial. A cut-off time of 300 s was set as complete memory recall (Azizbeigi et al., 2011; Zarrindast et al., 2012).

### 2.6. Western blotting

Following completion of the IA test on day 7 of the i.c.v. injection, four rats from each group were anesthetized, and the bilateral hippocampi were dissected to evaluate BDNF, TrkB, phosphorylated TrKB, CREB, and phosphorylated CREB protein levels by western blotting. The isolated hippocampal tissues were submerged in RIPA lysis buffer with a protease inhibitor cocktail (Abcam, United States) and homogenized using an ultrasonic homogenizer (FAPAN300; Fanavari Iranian Pajohesh Nassir, Iran). Following centrifugation at 13000 *g* for 10 min at 4°C, the supernatants were collected and the Bradford technique was used to measure protein concentrations (Bradford, 1976). Protein samples (20 μg per lane) were separated by 10% SDS-PAGE and transferred onto polyvinylidene difluoride (PVDF) membranes. After blocking in 2% non-fat dry milk for 75 min at room temperature (RT), the membrane was incubated with the following diluted primary antibodies (1:1000): anti-β-actin (SC-47778), anti-BDNF (Abcam-ab108319), anti-TrkB (SC-377218), anti-phosphorylated TrKB (anti-pTrKB, orb99306), anti-CREB (SC-377154), and anti-phosphorylated CREB (anti-pCREB, ab32096) at 4°C overnight. After washing the membranes in TBST buffer (Tris-buffered saline containing 0.1% Tween 20), they were incubated with appropriate secondary antibody conjugated with horseradish peroxidase (HRP, SC-2357, Santa Cruz, diluted 1:2000) at RT for 75 min. Immunoreactive protein bands were detected using enhanced chemiluminescence (ECL) reagents (Santa Cruz Biotechnology, United States). A mild stripping method was used to remove primary and secondary antibodies from the western blot membrane by using a stripping buffer (25 mM glycine-HCl, 1% SDS, pH adjusted to 2.2) before reprobing. Band densities were converted to surface area numbers by using ImageJ software (Ahmadi and Khaledi, 2020; Hashemi and Ahmadi, 2023).

### 2.7. Histological assessment

Nissl staining was performed to investigate the neuroprotective effects of APN treatment in the CA1 region (*n* = 4 per rat). On day 7 of the i.c.v. injection (day 21 of the schedule), four rats from each group were deeply anesthetized and perfused with 0.9% sodium chloride followed by a fixative solution through the left ventricle, including 4% paraformaldehyde in 0.1 M phosphate-buffered saline (pH 7.4). Whole brains were separated from the skull and fixed in the same fixative at 4°C overnight followed by paraffin embedding. Five-micrometer- sections were cut using a rotary microtome (Did Sabz Co., Urmia, Iran), and six hippocampal sections from each rat brain were mounted on glass slides. The slides were then dehydrated in graded ethanol solutions followed by Nissl staining using 0.1% cresyl violet for 2 min. The number of living cells in a small area of CA1 (15 * 10^3^ μm^2^) was counted in six sections per rat using ImageJ software. The average number of live neurons in six sections per rat was used for statistical analyses.

### 2.8. Statistical analysis

All data were presented as the mean ± SD. The differences among all experimental groups were compared and analyzed by one-way ANOVA followed by paired group comparisons using Tukey’s *post hoc* test. Statistical significance was set at *p* < 0.05. Statistical analyses were performed using the GraphPad Prism software package version 9.0. The corresponding author agrees to make data supporting the findings of this paper available upon reasonable request.

## 3. Results

### 3.1. APN treatment improved spatial working memory performance in a Y Maze task impaired by KA

**Figure 1:**
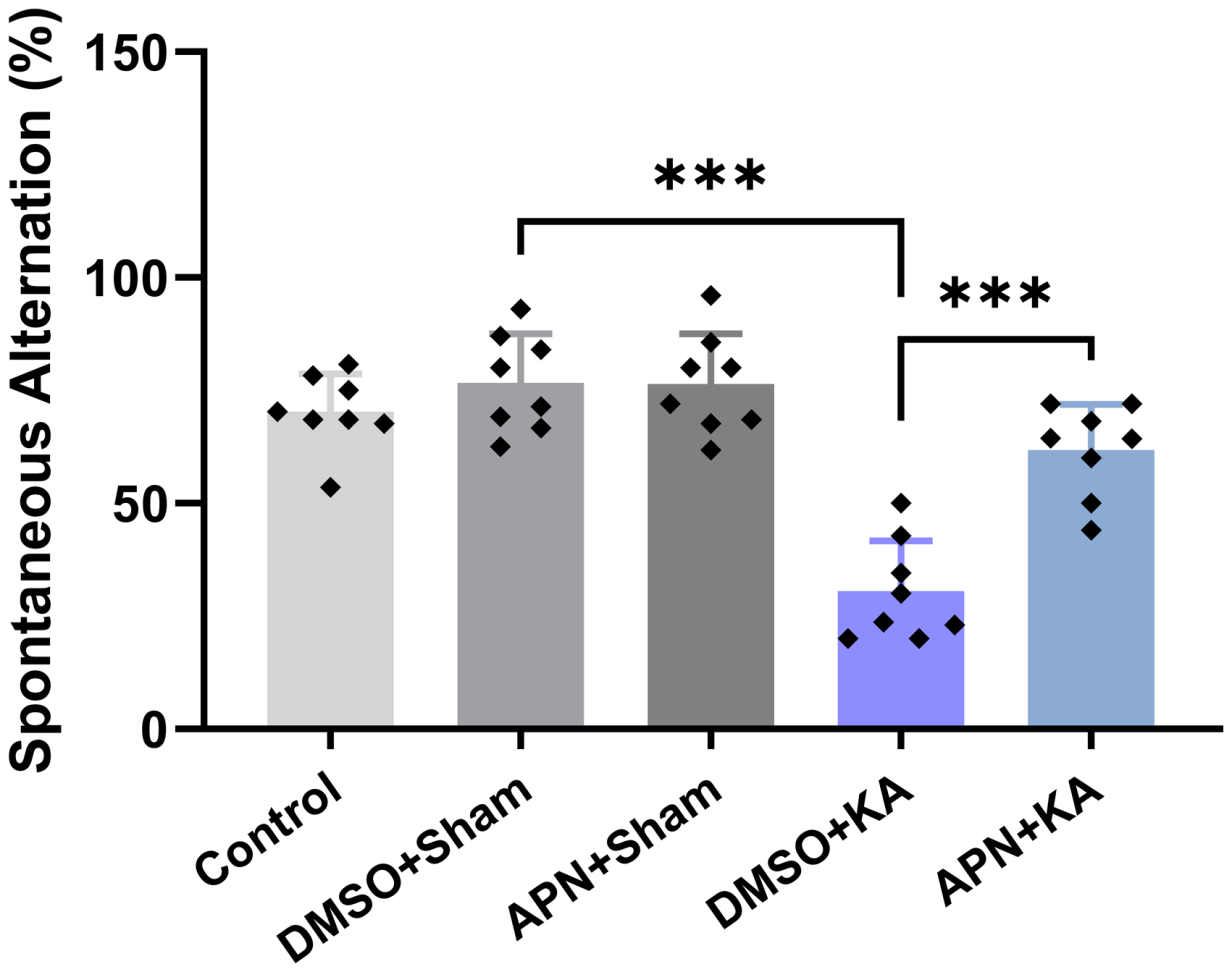
Effect of APN pre-and post-treatment (50 mg/kg/day) on spontaneous alternation behavior in a Y Maze task. All data are presented as the mean ± SD (*n* = 8 per experimental group). One-way ANOVA was used to identify the overall differences between the groups. ***p < 0.001: the significant statistical difference between the specified groups revealed by *post hoc* Tukey’s test.

Figure 1 shows the results of spatial working memory performance in the different experimental groups. One-way ANOVA revealed that the spontaneous alternation percentage was significantly different between the experimental groups [*F*(4, 35) = 27.5, *p* < 0.001]. *Post-hoc* Tukey’s test revealed that the spontaneous alternation percentage significantly decreased in the KA-treated group compared to those in the control, DMSO + Sham, and APN + Sham groups (*p* < 0.001). On the other hand, the decrease in the spontaneous alternation percentage induced by KA was significantly diminished following pre-and post-treatment of APN in the APN + KA group (*p* < 0.001).

### 3.2. APN treatment moderated the impairment of IA memory induced by KA in rats

**Figure 2:**
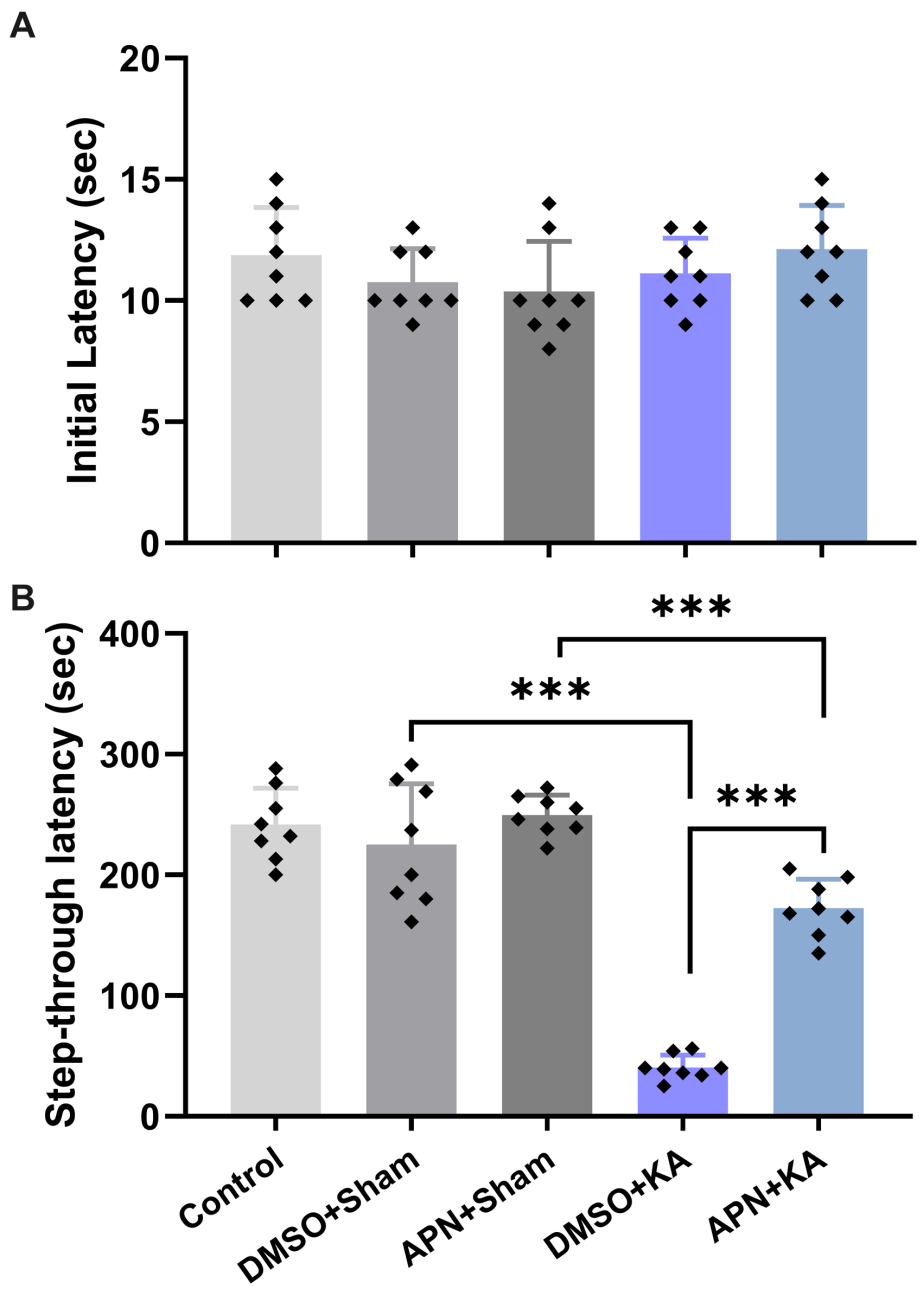
Effects of APN pre-and post-treatment (50 mg/kg/day) on IA memory performance. **(A)** Initial latencies in the acquisition phase and **(B)** step-through latencies in the memory-recall session. All data are presented as the mean ± SD (*n* = 8 per group). The overall difference between groups was determined using one-way ANOVA. The *post hoc* Tukey’s test indicated a statistically significant difference between the specified groups: ***p < 0.001.

Based on the results of the one-way ANOVA, no significant difference was detected in the initial latency between the experimental groups on the training day of the IA test [*F*(4, 35) = 1.41, *p* > 0.05]. However, a significant difference in step-through latency between groups was detected on the testing day of the IA test, suggesting significant changes in IA memory performance among the experimental groups [*F*(4, 35) = 68.73, *p* < 0.001]. Tukey’s *post hoc* analyses indicated that KA significantly impaired IA memory performance in the DMSO + KA group compared to that in the DMSO + sham group (*p* < 0.001). Interestingly, the APN pre- and post-treatment significantly moderated the impairment of IA memory performance (*p* < 0.001) as revealed by an increase in step-through latency in the APN + KA group compared to the DMSO + KA group (Figure 2).

### 3.3. APN treatment prevented neuronal cell loss induced by KA in the CA1 region of the hippocampus

**Figure 3:**
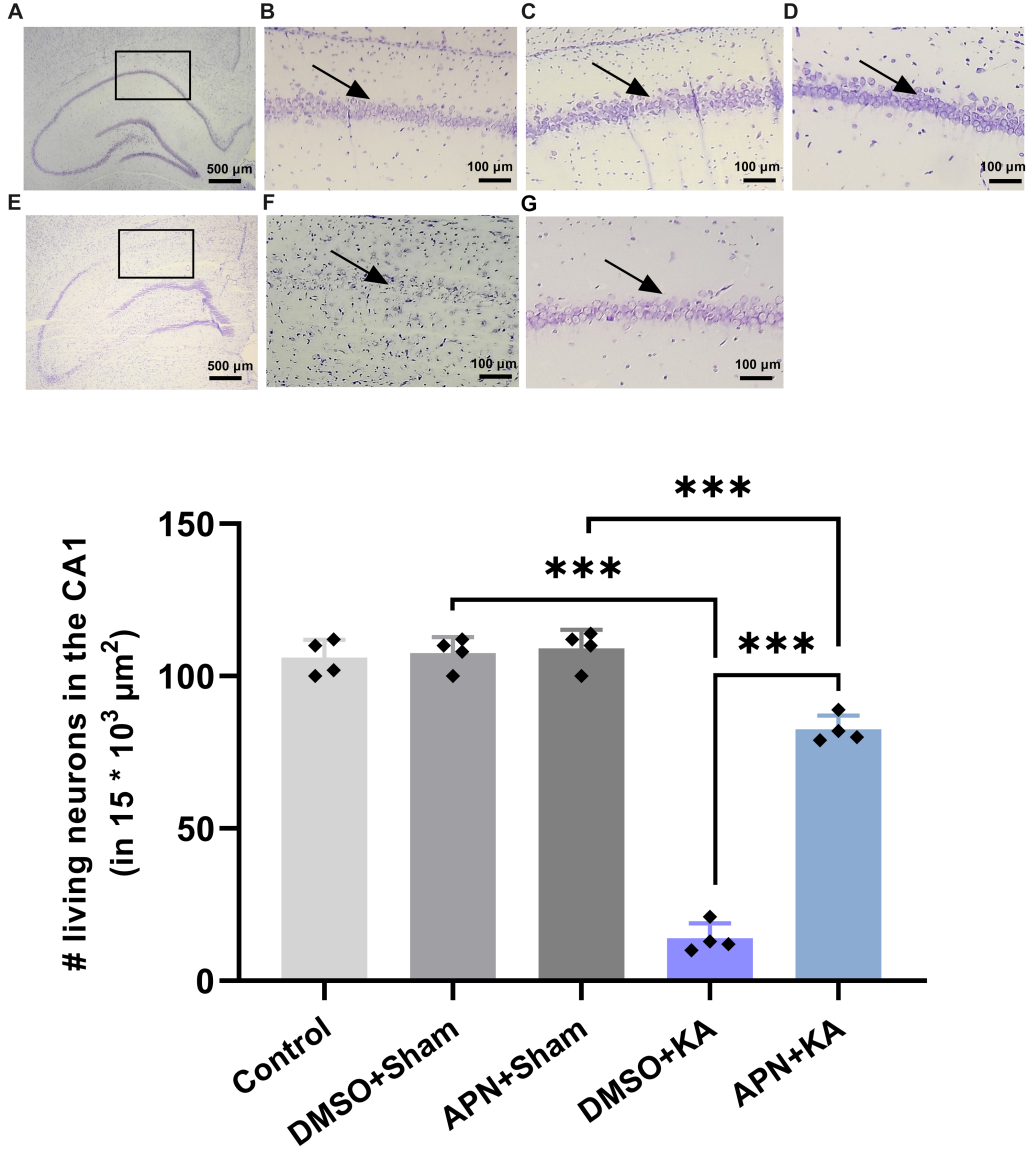
Neuroprotective effect of APN pre-and post-treatment (50 mg/kg/day) against neuronal cell loss in the CA1. The number of live neurons was counted in a small area of CA1 (15 * 10^3^  μm^2^) per CA1 section. **(A)** Is a coronal section of the whole healthy hippocampus, indicating the selected area for cell counting. Magnified photomicrographs represent magnified coronal sections of the CA1 in the experimental groups, including **(B)** control, **(C)** DMSO + sham, and **(D)** APN + sham groups. **(E)** Is a coronal section of the whole hippocampus in a KA-treated rat visualizing the extent of neuron loss in different areas of the hippocampus. **(F,G)** Are images of a magnified area of the CA1 in the DMSO + KA and APN + KA groups, respectively. Rectangles on images A and E represent the main part of the CA1. Arrows on some of the images indicate pyramidal cell layer in the CA1. The bar graph in the lower panel represents the quantification of the surviving neurons in the CA1 in each experimental group (*n* = 4 per group). Data are shown as the mean ± SD. One-way ANOVA was employed to assess the general difference between groups, and the subsequent *post hoc* Tukey’s test revealed a statistically significant difference among the specific groups: ***p < 0.001.

It is obvious that i.c.v. injections of KA induce neuronal cell death in the hippocampus of mice and rats (Jin et al., 2009; Hashemi and Ahmadi, 2023). In the current study, following Nissl staining, the number of live neurons in the CA1 region was counted for all experimental groups. The results of one-way ANOVA analyses revealed substantial differences between groups in the number of living cells in CA1 [*F*(4, 15) = 226.8, *p* < 0.001]. Paired group comparisons confirmed that i.c.v. microinjection of KA led to notable neuronal loss in the CA1 area compared to the control and both DMSO + sham and APN + sham groups (*p* < 0.001). Interestingly, APN pre-and post-treatment significantly decreased neuronal cell loss in the CA1 area induced by KA (*p* < 0.001) compared to the DMSO + KA group, suggesting a neuroprotective role for APN. However, the number of the CA1 living cells in the APN + KA group was lower than APN + sham group (Figure 3).

### 3.4. APN treatment increased hippocampal levels of BDNF, TrKB, and p-TrKB in a rat model of memory impairment induced by KA

**Figure 4:**
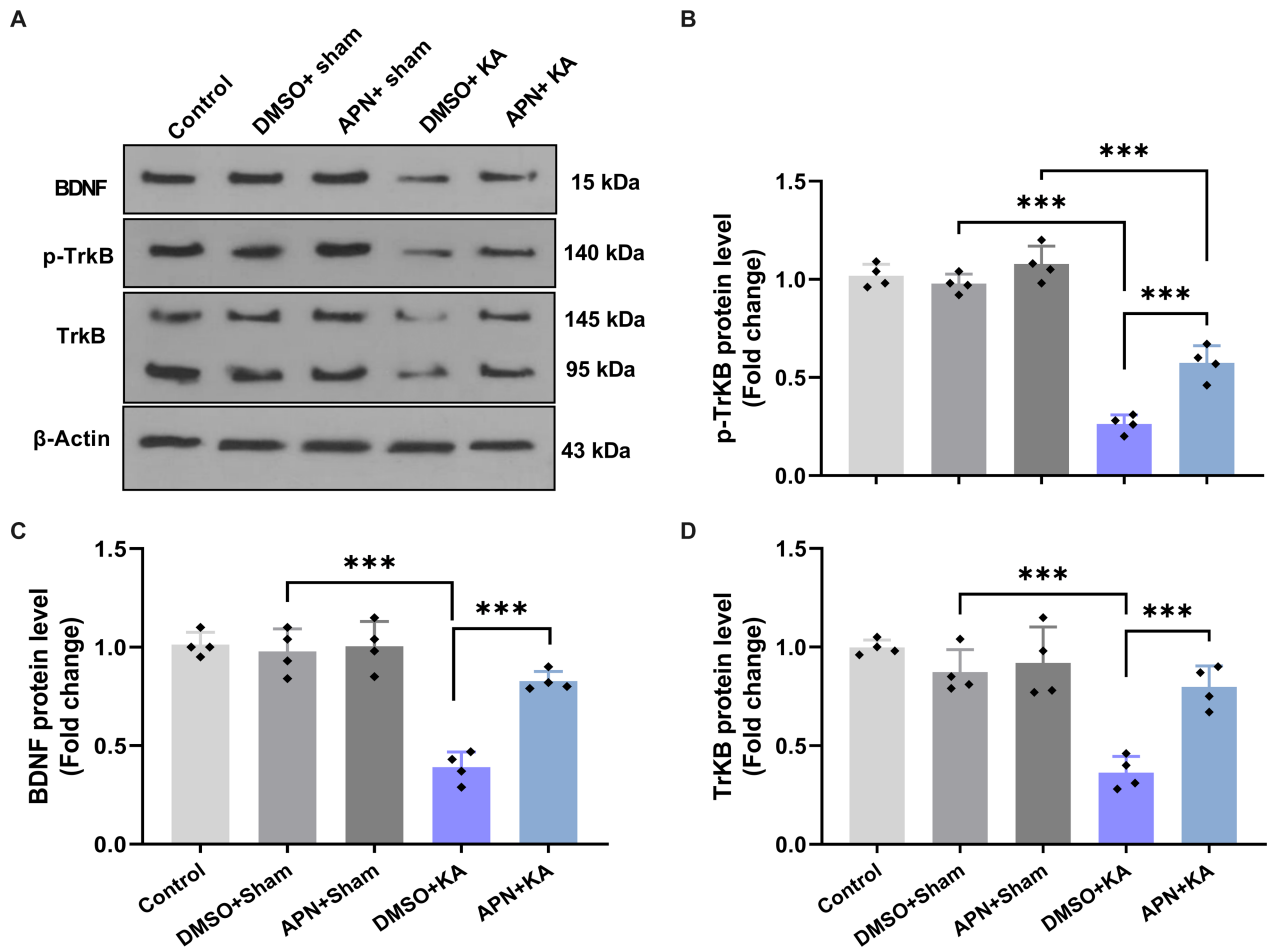
Effect of APN pre-and post-treatment (50 mg/kg/day) on hippocampal levels of BDNF, TrKB, and p-TrKB. **(A)** Western blotting images indicating band densities for BDNF, TrKB, and p-TrKB in the five experimental groups. β-actin was used as an internal reference. **(B–D)** Bar graphs represent the quantified protein levels of BDNF, TrkB, and p-TrkB in the hippocampus in each experimental group (*n* = 4 per group). All data are presented as the mean ± SD. The overall difference between groups was determined using one-way ANOVA. The *post hoc* Tukey’s test indicated a statistically significant difference between the specified groups: ***p < 0.001.

To examine the molecular mechanisms underlying the neuroprotective role of APN, the protein levels of BDNF and its receptor TrKB were examined in the hippocampus after 21 days of APN treatment. Western blot analysis of BDNF, TrKB, and p-TrKB protein levels in the hippocampus revealed significant differences between experimental groups for BDNF [*F*(4, 15) = 33.5, *p* < 0.001], TrkB [*F*(4, 15) = 18.99; *p* < 0.001], and p-TrKB [*F*(4, 15) = 102.3; *p* < 0.001]. Tukey’s *post hoc* test indicated that there were significant decreases in the hippocampal levels of BDNF, TrKB, and p-TrKB in the DMSO + KA group compared to those in the control, DMSO + sham, and APN + sham groups (*p* < 0.001). The APN pre-and post-treatment moderated (*p* < 0.001) the reductions in hippocampal levels of BDNF, TrKB, and p-TrKB induced by the i.c.v. injection of KA (Figure 4).

### 3.5. APN treatment increased hippocampal levels of CREB and p-CREB in a rat model of memory impairment induced by KA

**Figure 5:**
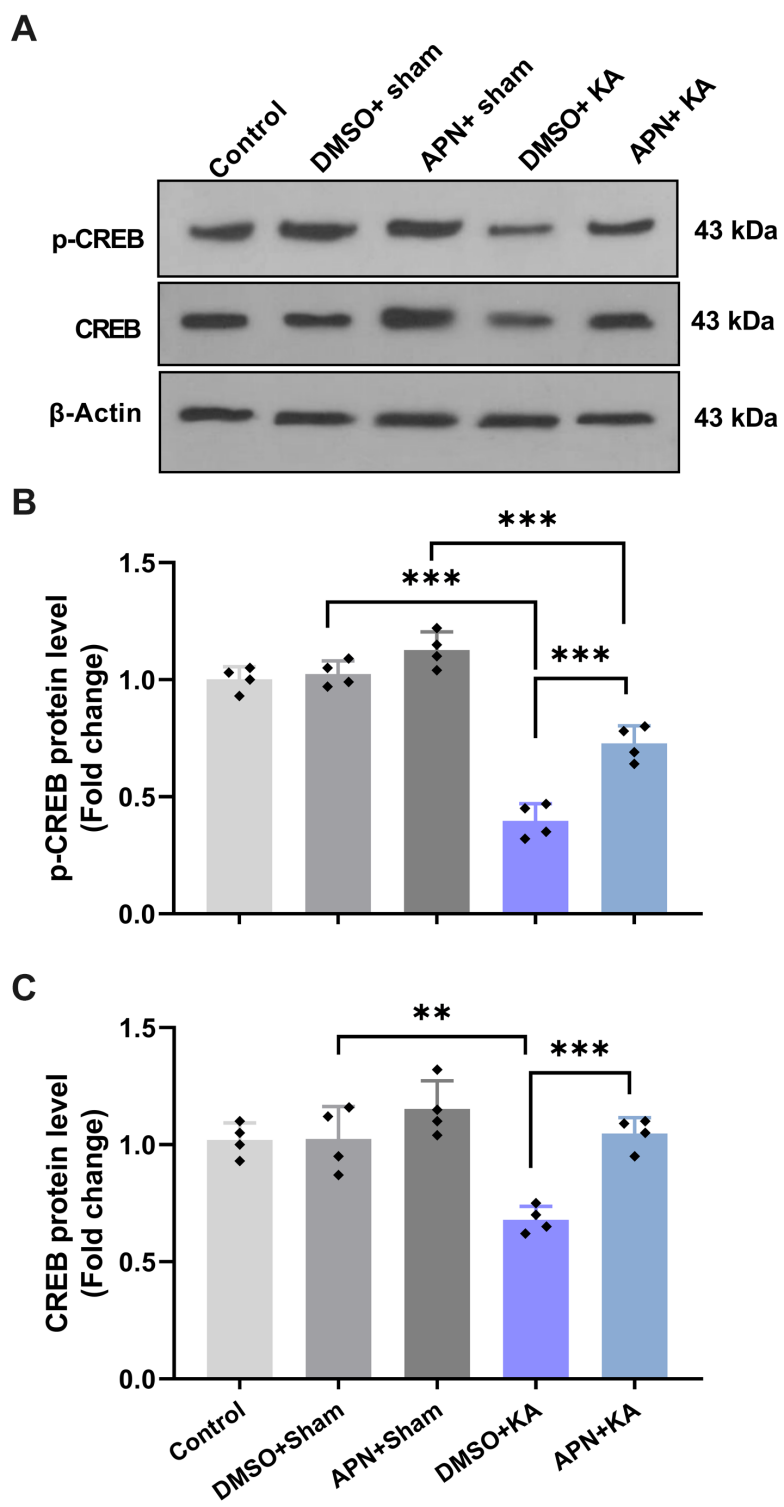
Effect of APN pre-and post-treatment (50 mg/kg/day) on CREB and p-CREB protein levels in the hippocampus. **(A)** Western blot images indicating CREB and p-CREB band densities in the five experimental groups. **(B, C)** Are bar graphs representing the quantified protein levels of CREB and p-CREB in the hippocampus in each experimental group (*n* = 4 per group). All data are presented as the mean ± SD. One-way ANOVA was employed to assess the general difference between groups, and the subsequent *post hoc* Tukey’s test revealed a statistically significant distinction among the specific groups: **p < 0.01 and ***p < 0.001.

According to research, different agents can improve learning and memory by increasing CREB activity (Sharma et al., 2019; Yan et al., 2022). We also examined the CREB and p-CREB protein levels in the hippocampus after 21 days of APN treatment. The results of western blotting for CREB and p-CREB protein levels in the hippocampus revealed significant differences between the experimental groups for both CREB [*F*(4, 15) = 13.69; *p* < 0.001] and p-CREB [*F*(4, 15) = 77.08; *p* < 0.001]. Paired group comparisons indicated that there were significant decreases in the hippocampal levels of CREB and p-CREB in the DMSO + KA group compared to those in the control, DMSO + sham, and APN + sham groups (*p* < 0.001). However, pre-and post-treatment of APN at a dose of 50 mg/kg partially prevented (*p* < 0.001) the reductions in CREB and p-CREB protein levels in the hippocampus induced by i.c.v. injection of KA (Figure 5).

## 4. Discussion

This study examined how the administration of APN before and after KA exposure affected learning and memory deficits in rats. To assess these effects, both the Y-maze and step-through IA tasks were employed. The levels of BDNF, TrKB, p-TrKB, CREB, and p-CREB were measured in the hippocampus, along with the evaluation of neuronal cell loss in that brain region. The results of the Y-maze test revealed that i.c.v. injection of KA impaired spatial memory performance in rats, which was attenuated by APN pre-and post-treatment. The Y Maze task is a well-known test for evaluating hippocampal-dependent spatial working memory in rodents (Kraeuter et al., 2019). Consistent with the present results, previous evidence has also documented that the injection of KA into different parts of the brain, including the lateral ventricle and hippocampus, leads to learning and memory impairments (Gordon et al., 2013; Khodamoradi et al., 2016). There was also a significant decrease in IA memory performance in the DMSO + KA group compared with that in the DMSO + sham group. However, pre-and post-treatment with APN remarkably improved IA memory performance in the APN + KA group compared to those in the DMSO + KA group. In addition, there was a group difference in IA memory performance between the APN + KA and APN + sham groups, which may imply that the APN pre-and post-treatment did not completely prevent memory impairment induced by KA. Extending the duration of the APN treatment is a suggestion to obtain a better memory-improving effect with APN; however, further studies are needed. Together, the results of the behavioral tests in the present study confirmed that APN pre-and post-treatment partially had preventive effects against KA-induced memory impairment.

Furthermore, the beneficial effect of APN on learning and memory deficits in animal models of neurodegenerative diseases has been previously reported (Goudarzi and Rafieirad, 2017; Lee et al., 2017). In a study, Lee et al. (2017) demonstrated that daily APN pretreatment mitigated memory deficit induced by scopolamine, probably via inhibiting oxidative stress and increasing the synthesis of acetylcholine (Lee et al., 2017). APN also has preventive effects against IA memory impairment by regulating antioxidative and anti-acetylcholinesterase mechanisms, as well as enhancing dopamine concentration in a rat model of Parkinson’s disease (Goudarzi and Rafieirad, 2017). Other studies have revealed that APN not only improves memory deficits in amnesia-related models but also enhances learning and memory abilities in normal rats (Nozari and Rafieirad, 2019; Ahmadi-Kanali et al., 2021). In this regard, it has been shown that intrahippocampal injection of APN increases spatial memory by reducing the time latency and total distance traveled to reach the hidden platform in the acquisition phase of the Morris water maze (MWM) test and improves IA memory performance (Ahmadi-Kanali et al., 2021). Moreover, it has been reported that APN administration for 2 weeks significantly enhanced IA memory performance due to decreased MDA levels and increased thiol concentration in the hippocampus (Nozari and Rafieirad, 2019). However, the results of the current study indicated that APN pre-and post-treatment by itself had no enhancing effect on learning and memory performance in the sham-operated group compared to the control group. Differences in methodology, including the drug doses and route of drug administration, may account for the discrepancy between other reports and the results of the current research on the effect of APN on memory performance.

Hippocampal sclerosis is the main cause of memory complications following KA injection and is defined by massive neurodegeneration, particularly in the CA1, CA3, and hilar region (CA4) of the hippocampus (Malmgren and Thom, 2012). There are direct correlations between memory dysfunction and neuronal damage in the hippocampus (Miltiadous et al., 2011). Accumulating evidence indicates that KA administration into the lateral ventricle causes neurodegeneration within the hippocampus, which is accompanied by learning and memory deficits in rodents (Hashemi et al., 2019). The current findings demonstrate a wide range of neuronal cell loss in the CA1 region of the hippocampus due to the microinjection of KA into the left lateral ventricle. Interestingly, APN pre-and post-treatment at a dose of 50 mg/kg for 21 days significantly moderated KA-induced neuronal degeneration in the CA1. However, there was still a significant group difference in the number of CA1 surviving neurons between the APN + KA group and the APN + sham group, indicating some neuronal cell loss due to KA injection, even in the presence of APN pre-and post-treatment. Our findings are consistent with a recent study indicating that APN reverses amyloid-beta-induced neuronal cell loss by increasing the number of CA1 neurons in male rats (Khan-Mohammadi-Khorrami et al., 2020). We have also recently shown that APN pre-and post-treatment for 19 days has a neuroprotective effect against neuronal cell loss induced by i.c.v. injection of KA in the CA3 and CA4 of the hippocampus. Our previous data indicated that the neuroprotective effect of APN pre-and post-treatment is mediated by preventing the activation of the apoptotic pathway in the hippocampus (Hashemi and Ahmadi, 2023). It seems that the beneficial effects of APN on KA-induced learning and memory impairment also result from its neuroprotective effects in the hippocampus.

APN is a small and lipophilic molecule; therefore, it can cross cellular membranes, taking advantage of the natural permeability of membranes to small non-polar molecules (Yang and Hinner, 2015). Animal studies have also shown that APN penetrates the blood–brain barrier 30 min after inhalation (Satou et al., 2013, 2017). Similar to other terpenes, APN can interact with various targets including different membrane receptors, intracellular receptors, enzymes, and other cellular components to exert its pharmacological effects (Liktor-Busa et al., 2021). These effects include antioxidant, anti-inflammatory, and neuroprotective activities, as reported by different investigators. Research has revealed that the memory-improving effect of alpha-pinene may be mediated by its effect on cholinergic neurotransmission. In particular, APN has a therapeutic effect on Alzheimer’s disease by inhibiting acetylcholine esterase, an enzyme involved in the breakdown of acetylcholine (Wojtunik-Kulesza et al., 2021). APN treatment also increases the mRNA expression of choline acetyltransferase, an enzyme that catalyzes the production of acetylcholine (Lee et al., 2017). In addition, research has shown that APN acts as a partial modulator of GABA_A_-benzodiazepine receptors and enhances GABAergic synaptic transmission directly by binding to the benzodiazepine-binding site of the GABA_A_ receptor (Yang et al., 2016; Rafie et al., 2022). However, the exact molecular mechanisms underlying APN action remain unclear and require further investigations.

Numerous investigations have confirmed that among all neurotrophins, BDNF and its receptor TrKB play an essential role in adult synaptic plasticity, learning, and memory formation (Andero et al., 2014; Lu et al., 2014). Accordingly, BDNF deficiency has been associated with memory and cognitive impairments in neurodegenerative diseases, including Alzheimer’s disease (Amidfar et al., 2020), and also with memory deficits in TLE (de Almeida et al., 2017). It has been reported that the downregulation of TrkB in the hippocampus is associated with the progression of Alzheimer’s disease and subsequent memory decline (Ginsberg et al., 2019). To investigate the molecular mechanisms underlying the improving effects of pre-and post-treatment of APN on memory performance, we assessed the protein levels of BDNF and its receptor, TrkB, as well as CREB in the rat hippocampus following 21 days of the APN treatment. According to our findings, hippocampal levels of BDNF, TrkB, and phosphorylated TrKB (p-TrKB) were remarkably decreased in the DMSO + KA group compared to those in the DMSO + sham group. In agreement with these results, studies have also demonstrated diminished BDNF levels in the hippocampus following KA administration (Şahin et al., 2019). Interestingly, the results of the present experiments indicated that pre-and post-treatment with APN significantly moderated the KA-induced decreases in the hippocampal levels of BDNF, TrkB, and p-TrkB.

In support of the current results, it has been reported that inhalation of APN stimulates BDNF expression in the hippocampus of mice (Kasuya et al., 2015). There are some reports that astrocytic BDNF and TrkB molecules in the hippocampus are promising therapeutic targets for the treatment of TLE (Fernández-García et al., 2020). We have also recently reported that APN pre-and post-treatment protects hippocampal neurons via, at least partly, inhibiting pro-apoptotic Bax and increasing anti-apoptotic Bcl2 proteins in the hippocampus (Hashemi and Ahmadi, 2023). The present results indicate that APN treatment also increases BDNF and TrKB levels, supporting the neuroprotective effects of APN in the hippocampus. BDNF plays a crucial role in the development, growth, survival, and function of neurons in developing and adult mammalian brains. When BDNF binds to the TrkB receptor, it triggers phosphorylation of the receptor and subsequently a series of intracellular signaling pathways. TrkB receptors dimerize following BDNF binding, leading to transphosphorylation of the autophosphorylation loops at tyrosine residues 705/6 (Y705/6) and phosphorylation of tyrosine residues 515 and 816 (Y515 and 816), which initiate downstream signaling cascades, including the mitogen-activated protein kinase (MAPK) and phosphatidylinositol 3-kinase (PI3K) pathways, transmitting the signal to the nucleus (Minichiello et al., 1999; Minichiello, 2009; Numakawa et al., 2010). Activated signaling pathways induce changes in gene expression within the target cell nucleus, including increased expression of BDNF and the TrkB receptor. This upregulation of TrkB receptors enhances cell sensitivity and responsiveness to BDNF signaling, facilitating the important functions of BDNF in neuronal growth, survival, and plasticity (Alonso et al., 2002; Deinhardt and Chao, 2014; Leal et al., 2014).

Moreover, the protective impact of APN has been evidenced in numerous investigations [for review see (Weston-Green et al., 2021)]. It has been shown that APN modulates BDNF and its receptor TrkB in certain contexts. Kasuya et al. (2015) reported an increase in mRNA levels of BDNF in the olfactory bulb and hippocampus following inhaled administration of APN (Kasuya et al., 2015). However, the exact mechanisms underlying the effects of APN on BDNF and TrkB are still being studied and further research is needed to fully understand the underlying processes. APN can potentially influence the activity of transcription factors, such as CREB, which bind to specific regions of the BDNF gene, promoting its transcription. This increased transcription of BDNF leads to higher levels of BDNF protein production in the neurons. APN may also enhance the binding affinity between BDNF and TrkB, leading to increased activation of TrkB receptors and resulting in enhanced cellular responses mediated by BDNF–TrkB signaling. BDNF levels have been suggested as a valuable indicator of cognitive states (Amidfar et al., 2020). Therefore, the increases in hippocampal BDNF and TrKB levels following APN treatment validate the positive effects of APN on memory performance.

CREB, a key transcription factor downstream of many intracellular signaling pathways, mainly regulates the expression of molecules involved in memory functions, including BDNF (Josselyn and Nguyen, 2005; Amidfar et al., 2020). A reduction in CREB expression has also been associated with memory impairment in a mouse model of TLE induced by pilocarpine (Xing et al., 2019). We also examined hippocampal CREB levels in all experimental groups to evaluate changes compared to BDNF levels. According to the present results, KA injection decreased hippocampal levels of CREB and phosphorylated CREB (p-CREB), which was partially rescued by APN pre-and post-treatment. Considering the effects of APN on hippocampal levels of CREB and BDNF, we reasoned that the increased levels of CREB and p-CREB are a possible mechanism for the improving effects of APN on hippocampal BDNF levels and memory performance in the Y-maze and IA tasks. Other investigators have also reported that plant-derived compounds improve memory functions in animal models of Alzheimer’s disease via increasing CREB and BDNF (Pak et al., 2022; Yan et al., 2022). Together, these data suggest that the BDNF/TrKB/CREB signaling pathway may account for the improved effects of APN treatment on KA-induced memory impairment.

Several pathways are involved in CREB activation, including receptor tyrosine kinases, G protein-coupled receptors, and ionotropic receptors (Shaywitz and Greenberg, 1999; Mizuno et al., 2002). It has been reported that the pharmacological effects of APN are mediated by its interaction with various target molecules on either the cell membrane or inside the cells (Liktor-Busa et al., 2021). Considering the involvement of BDNF/TrKB and CREB activation in learning and memory processes and the increase in these molecules due to APN treatment in the present study, we hypothesized that APN leads to CREB activation by increasing BDNF/TrKB signaling pathway. However, CREB activation via other signaling cascades following APN treatment cannot be excluded and requires further experiments.

## 5. Conclusion

Two potential explanations for these findings can be put forward. First, it is plausible that the decrease in BDNF, p-TrKB/TrKB, and p-CREB/CREB proteins be a consequence of neuronal death in the hippocampus following KA injection. Second, decreases in BDNF, p-TrKB/TrKB, and p-CREB/CREB proteins play a causal role in neuronal death in the hippocampus following KA injection. Both of the two possibilities should be considered. The hippocampus is particularly vulnerable to KA-induced cytotoxicity and neuronal damage. When neurons die, normal protein expression and signaling processes are disrupted. Therefore, the decrease in BDNF, p-TrKB/TrKB, and p-CREB/CREB protein levels in the hippocampus reflect, on one hand, the damage and subsequent death of neurons, which leads to a reduction in their expression levels. On the other hand, BDNF plays a crucial role in supporting neuronal survival and protecting against neurodegenerative processes by employing various protective mechanisms (Almeida et al., 2005; Chen et al., 2017). BDNF can inhibit apoptotic cell death by counteracting KA-induced pro-apoptotic signals (Chiu et al., 2019). BDNF and TrKB neurotrophic signaling also enhance neuronal survival by supporting survival signaling cascades within neurons (Rössler et al., 2004; Wang et al., 2019). In addition to its well-established neurotrophic action, BDNF also possesses other neuroprotective effects including anti-apoptosis, anti-oxidation, and autophagy suppression (Chen et al., 2017). These mechanisms collectively contribute to BDNF’s ability to support neuronal survival in the hippocampi of KA-treated animals. Therefore, increases in BDNF, p-TrKB/TrKB, and p-CREB/CREB proteins and prevention of neuronal death in the hippocampus due to APN treatment support the suggestion that the hippocampus is particularly vulnerable to KA-induced damage due to blocking BDNF signaling. We have recently shown that APN can modulate the balance between pro-apoptotic and anti-apoptotic proteins in the hippocampus of animals treated with KA, thereby promoting cell survival (Hashemi and Ahmadi, 2023). Taken together, it can be concluded that APN can directly or indirectly employ various targets involved in cell survival such as BDNF/TrKB/CREB to exert neuroprotective effects against KA damage in the hippocampus.

## Data availability statement

The raw data supporting the conclusions of this article will be made available by the authors, without undue reservation.

## Ethics statement

The animal study was reviewed and approved by the Ethics and Research Committee at the University of Kurdistan (IR.UOK.REC.1400.024).

## Author contributions

PH: conceptualization and design of the work, acquisition, analysis, interpretation of data, and writing—original draft preparation. SA: conceptualization and design of the work, supervision, project administration, funding, and writing—reviewing and editing. All authors contributed to the article and approved the submitted version.

## Funding

This study was supported by the office of the Vice President for Research and Technology, University of Kurdistan (Grant no. 1399).

## Conflict of interest

The authors declare that the research was conducted in the absence of any commercial or financial relationships that could be construed as a potential conflict of interest.

## Publisher’s note

All claims expressed in this article are solely those of the authors and do not necessarily represent those of their affiliated organizations, or those of the publisher, the editors and the reviewers. Any product that may be evaluated in this article, or claim that may be made by its manufacturer, is not guaranteed or endorsed by the publisher.
