# Supplementary material for: Alpha-pinene moderates memory impairment induced by kainic acid via improving the BDNF/TrkB/CREB signaling pathway in rat hippocampus
Source: Front Mol Neurosci. 2023 Jun 30;16:1202232. doi: 10.3389/fnmol.2023.1202232 (PMC10347414; doi:10.3389/fnmol.2023.1202232)
Supplement: Supplementary file 1 [file Data_Sheet_1.PDF]

Supplementary Material

Alpha-pinene moderates memory impairment induced by kainic acid via improving the BDNF/TrkB/CREB signaling pathway in rat hippocampus

Paria Hashemi[1], Shamseddin Ahmadi[1*]

[1]Department of Biological Science, Faculty of Science, University of Kurdistan, Sanandaj, Iran

*** Correspondence:**

Shamseddin Ahmadi

sh.ahmadi@uok.ac.ir

Department of Biological Science, Faculty of Science, University of Kurdistan, P.O. Box 416, Sanandaj, Iran.

Tel: +98-87-33660075

Fax: +98-87-33622702

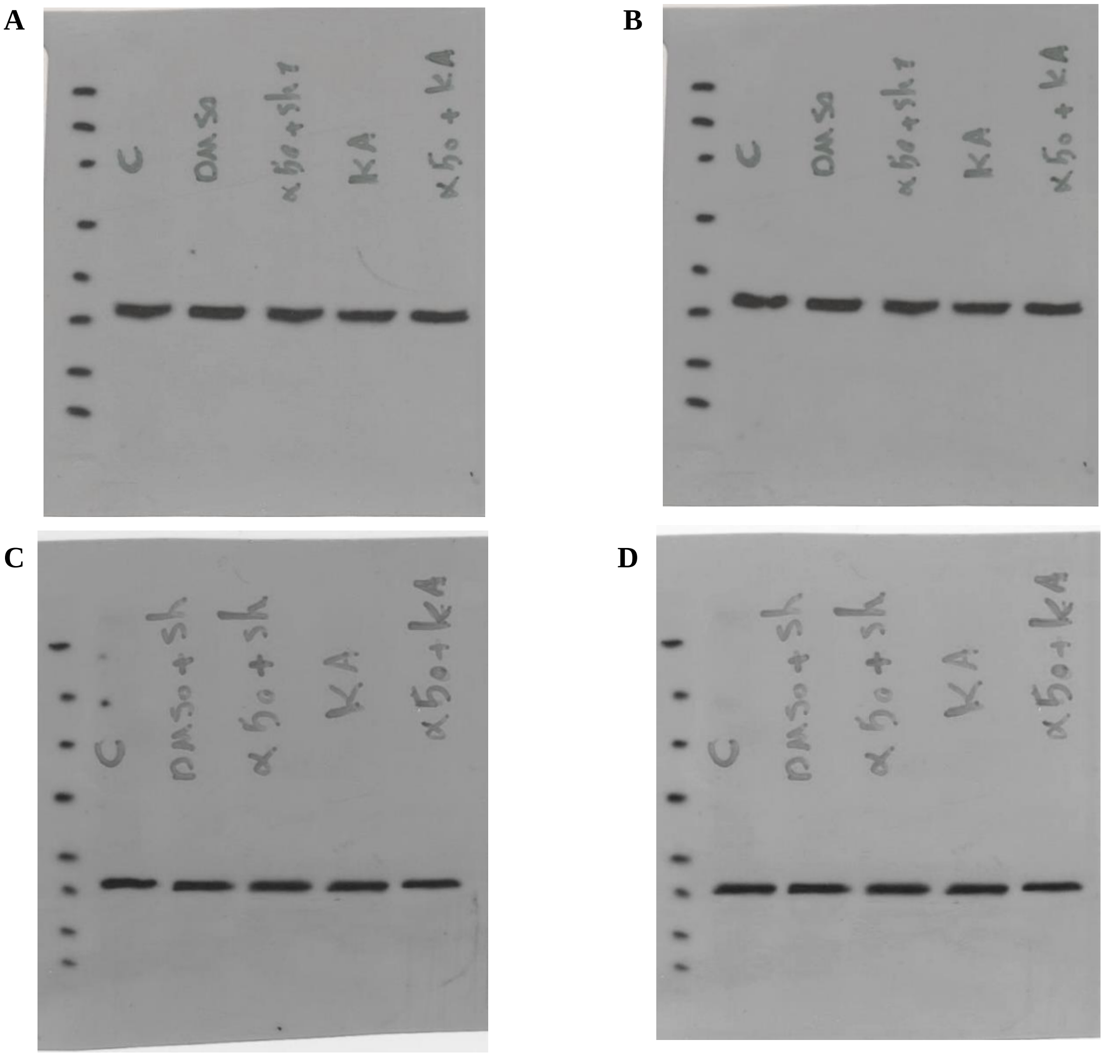

Supplementary Figure 1: Original images of western blot results indicating β-actin bands are shown in A, B, C, and D. Each image represents β-actin bands (43 kDa) corresponding to one biological repeat in all experimental groups. The protein bands, from left to right, display the protein marker and β-actin bands in the control, DMSO + Sham, APN + Sham, DMSO + KA, and APN + KA groups, respectively.

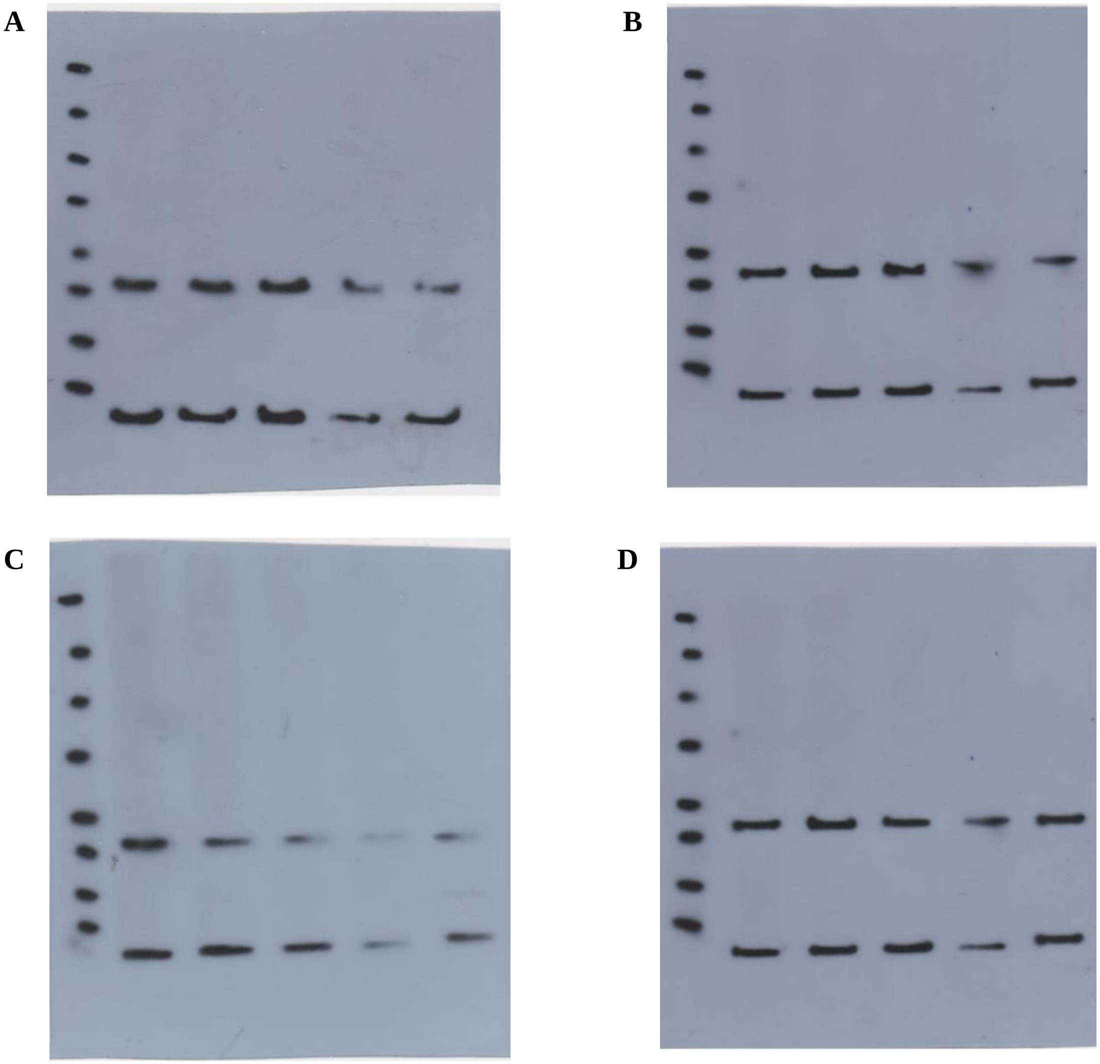

Supplementary Figure 2: Original images of western blot results indicating BDNF bands are shown in A, B, C, and D. Each image represents BDNF bands (15 and 45 kDa) corresponding to one biological repeat in all experimental groups. The protein bands, from left to right, display the protein marker and BDNF bands in the control, DMSO + Sham, APN + Sham, DMSO + KA, and APN + KA groups, respectively.

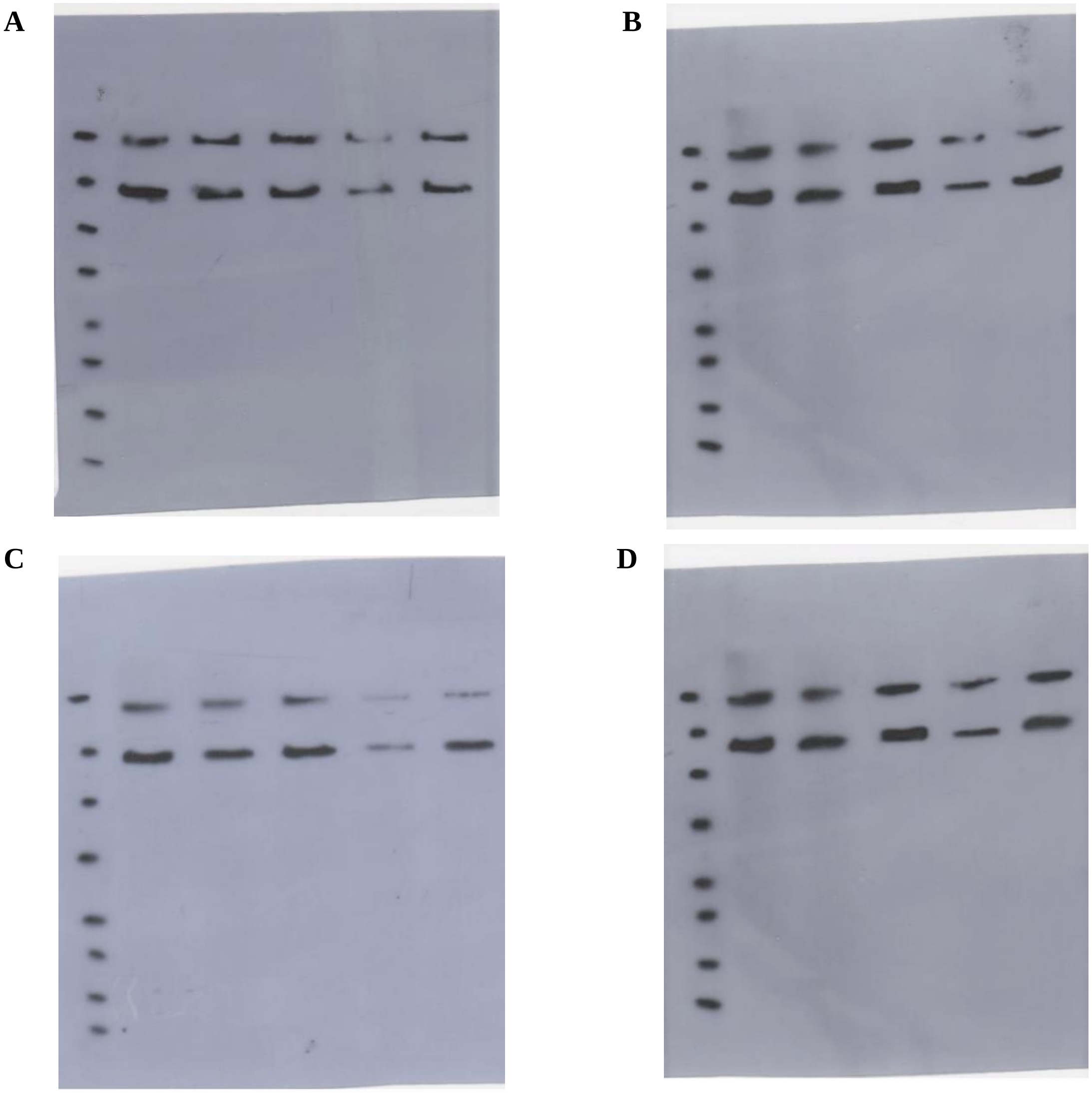

Supplementary Figure 3: Original images of western blot results indicating TrKB bands are shown in A, B, C, and D. Each image represents TrKB bands (95 and 145 kDa) corresponding to one biological repeat in all experimental groups. The protein bands, from left to right, display the protein marker and TrKB bands in the control, DMSO + Sham, APN + Sham, DMSO + KA, and APN + KA groups, respectively.

A

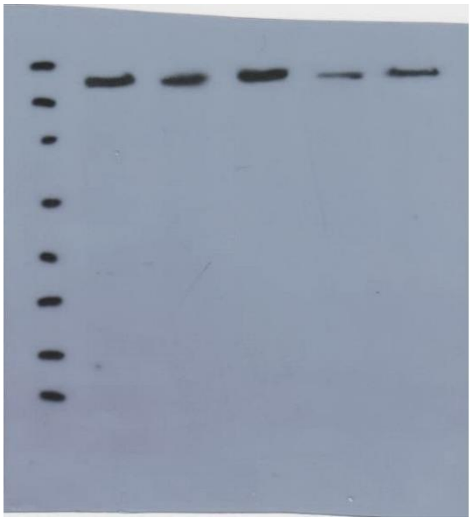

B

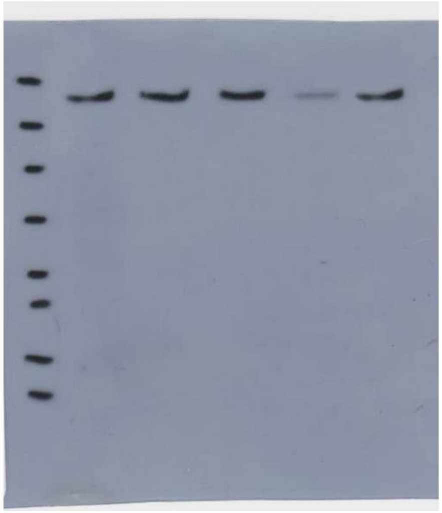

C

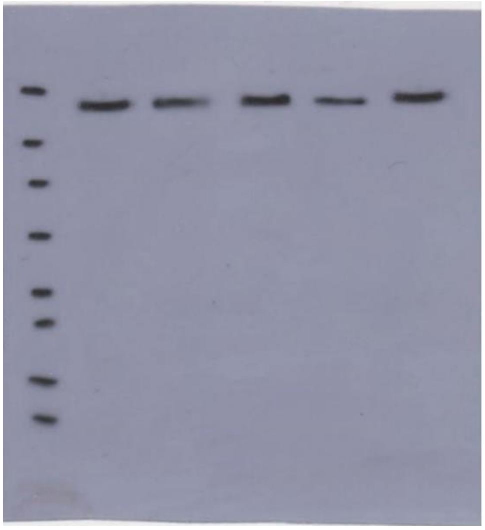

D

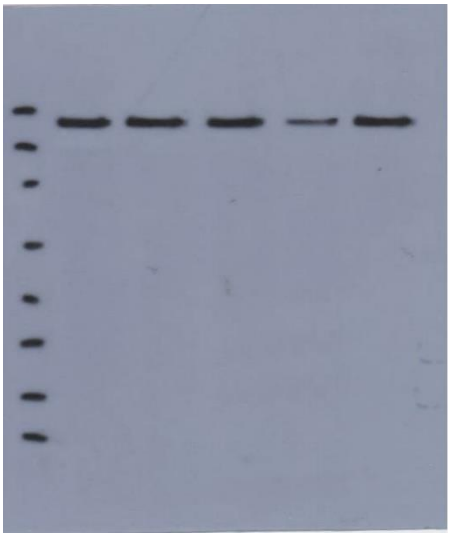

Supplementary Figure 4: Original images of western blot results indicating phospho-TrKB bands are shown in A, B, C, and D. Each image represents phospho-TrKB bands (140 kDa) corresponding to one biological repeat in all experimental groups. The protein bands, from left to right, display the protein marker and phospho-TrKB bands in the control, DMSO + Sham, APN + Sham, DMSO + KA, and APN + KA groups, respectively.

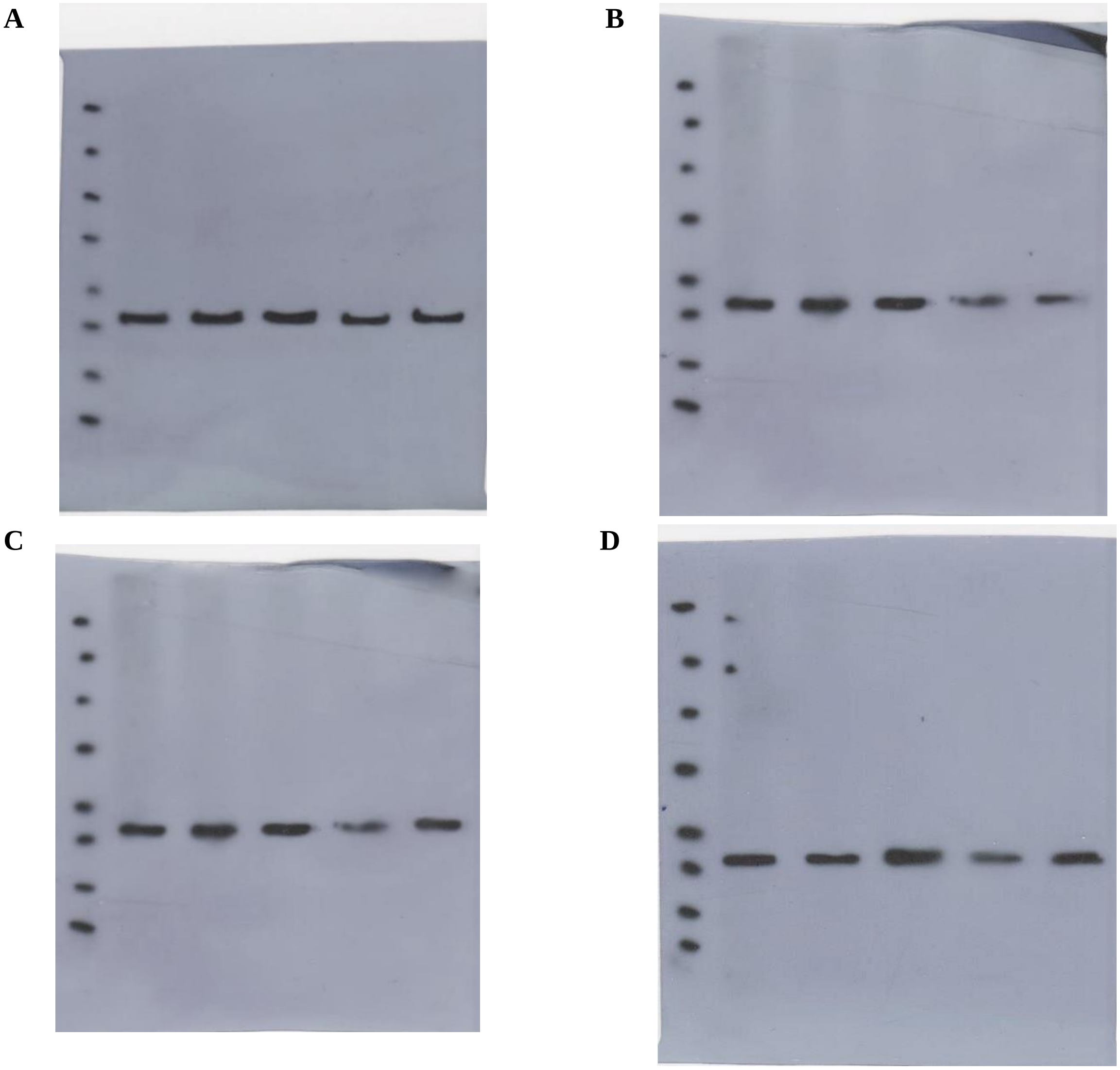

Supplementary Figure 5: Original images of western blot results indicating CREB-1 bands are shown in A, B, C, and D. Each image represents CREB-1 bands (43 kDa) corresponding to one biological repeat in all experimental groups. The protein bands, from left to right, display the protein marker and CREB-1 bands in the control, DMSO + Sham, APN + Sham, DMSO + KA, and APN + KA groups, respectively.

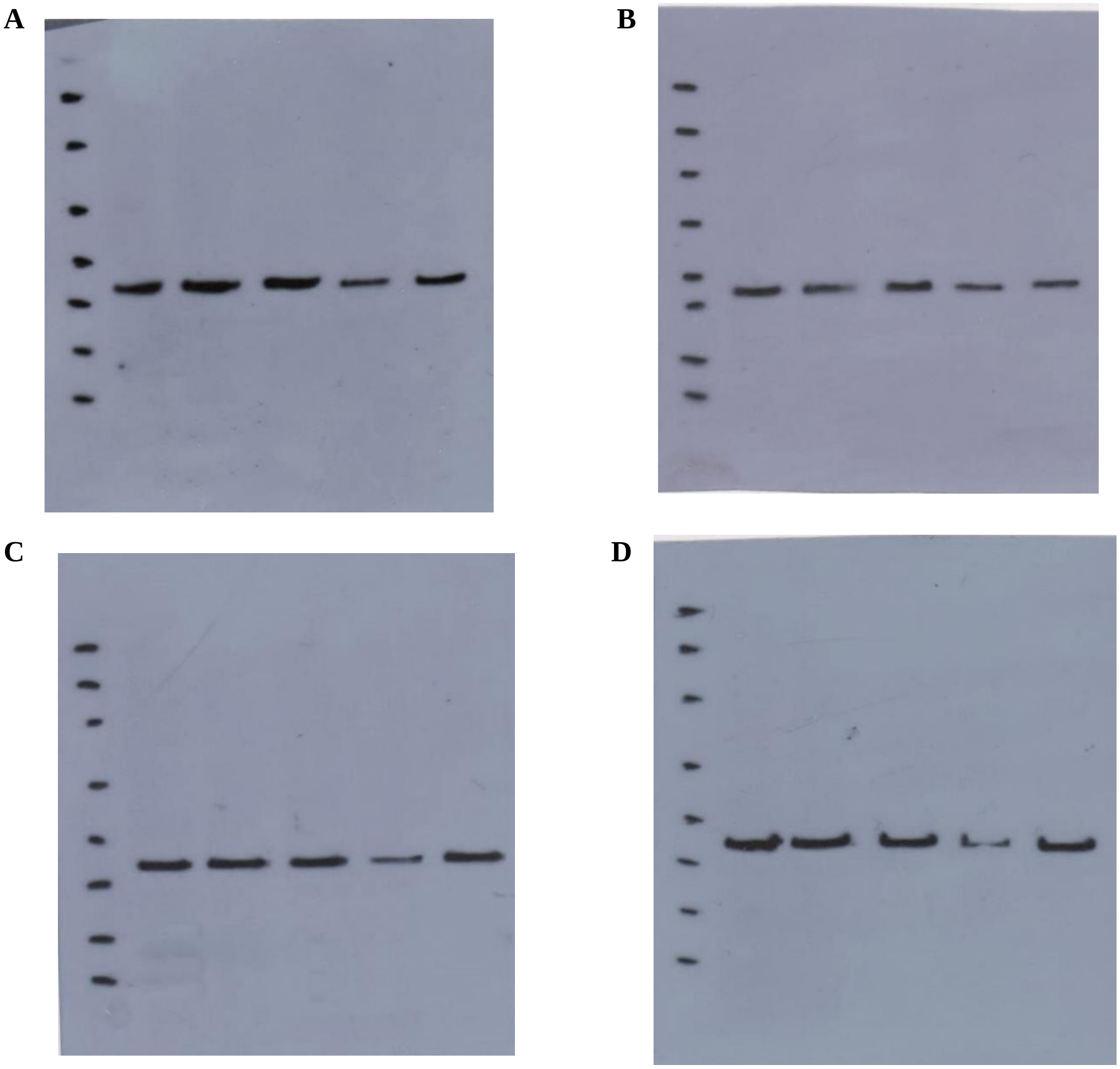

Supplementary Figure 6: Original images of western blot results indicating phospho-CREB bands are shown in A, B, C, and D. Each image represents phospho-CREB bands (43 kDa) corresponding to one biological repeat in all experimental groups. The protein bands, from left to right, display the protein marker and phospho- CREB bands in the control, DMSO + Sham, APN + Sham, DMSO + KA, and APN + KA groups, respectively.
